# Translation of robot-assisted rehabilitation to clinical service: a comparison of the rehabilitation effectiveness of EMG-driven robot hand assisted upper limb training in practical clinical service and in clinical trial with laboratory configuration for chronic stroke

**DOI:** 10.1186/s12938-018-0516-2

**Published:** 2018-06-25

**Authors:** Yanhuan Huang, Will Poyan Lai, Qiuyang Qian, Xiaoling Hu, Eric W. C. Tam, Yongping Zheng

**Affiliations:** 1 Department of Biomedical Engineering, Interdisciplinary Division of Biomedical Engineering, The Hong Kong Polytechnic University, Hong Kong, China; 2 Jockey Club Rehabilitation Engineering Clinic, Department of Biomedical Engineering, The Hong Kong Polytechnic University, Hong Kong, China

**Keywords:** Stroke, Upper limb, Rehabilitation, Robot, Clinical service

## Abstract

**Background:**

Rehabilitation robots can provide intensive physical training after stroke. However, variations of the rehabilitation effects in translation from well-controlled research studies to clinical services have not been well evaluated yet. This study aims to compare the rehabilitation effects of the upper limb training by an electromyography (EMG)-driven robotic hand achieved in a well-controlled research environment and in a practical clinical service.

**Methods:**

It was a non-randomized controlled trial, and thirty-two participants with chronic stroke were recruited either in the clinical service (n = 16, clinic group), or in the research setting (n = 16, lab group). Each participant received 20-session EMG-driven robotic hand assisted upper limb training. The training frequency (4 sessions/week) and the pace in a session were fixed for the lab group, while they were flexible (1–3 sessions/week) and adaptive for the clinic group. The training effects were evaluated before and after the treatment with clinical scores of the Fugl-Meyer Assessment (FMA), Action Research Arm Test (ARAT), Functional Independence Measure (FIM), and Modified Ashworth Scale (MAS).

**Results:**

Significant improvements in the FMA full score, shoulder/elbow and wrist/hand (P < 0.001), ARAT (P < 0.001), and MAS elbow (P < 0.05) were observed after the training for both groups. Significant improvements in the FIM (P < 0.05), MAS wrist (P < 0.001) and MAS hand (P < 0.05) were only obtained after the training in the clinic group. Compared with the lab group, higher FIM improvement in the clinic group was observed (P < 0.05).

**Conclusions:**

The functional improvements after the robotic hand training in the clinical service were comparable to the effectiveness achieved in the research setting, through flexible training schedules even with a lower training frequency every week. Higher independence in the daily living and a more effective release in muscle tones were achieved in the clinic group than the lab group.

## Background

Stroke is a major cause of permanent disability in adults [1]. By 2014, the number of stroke survivors in Hong Kong was approximately 300,000, and more than 7 million in Mainland China, with an average of 2 million new cases per year and an annual increase of 8% from 2009 to 2014 in Mainland China [2, 3]. Approximately 80% of stroke survivors experience upper extremity impairment and disability in activities of daily living (ADLs) [4, 5]. However, fewer than 25% of these can regain limited recovery on their paretic arms even after post-stroke rehabilitation [6]. Physical treatment can result in more significant recovery of arm function during the subacute period (i.e., before 6 months after stroke onset) than in the chronic stage (i.e., more than 6 months after the stroke onset) [7]. In current clinical practice, the professional manpower of post-stroke rehabilitation is much more concentrated on the in-patient period in the subacute stage, compared with that in the long-term service for chronic stroke. However, recent studies have demonstrated that with intensive training, significant motor improvements could also be achieved during the chronic period after stroke [8, 9]. The challenge, however, is that rehabilitation manpower is insufficient, even in developed countries with the fast-expanding stroke populations. Hence, effective techniques and services for long-term rehabilitation after stroke are in urgent need.

Rehabilitation robots have been valuable for human therapists in delivering the labor-demanding physical training with the advantages of higher repetition and lower cost than professional manpower [10]. Various robots have been proposed for the upper limb rehabilitation after stroke, and the robots’ effectiveness has been evaluated by clinical trials [11–13]. Among them, robot-assisted rehabilitation controlled by the voluntary inputs of the user exhibited more significant efficacy than that with continuous passive motions, i.e., no voluntary input was required from a user and the robots dominated the motion of a paralyzed limb [14]. In a voluntary intention driven robot designed by Song et al. [15], electromyography (EMG) from the residual muscle of the upper limb was used as the indicator of the voluntary motor intention from a stroke survivor. In the related randomized clinical trial, it was found that patients with chronic stroke obtained more significant motor gain when assisted with the EMG-driven robot than with passive motion assistance alone [16]. Another representative study was the large randomized multi-center trial by Lo et al. which compared the MIT-Manus robotic system for upper limb training with the conventional physical treatments by a human therapist [17]. The results suggested that the robot could achieve the equivalent motor improvements to those of the conventional treatment [17]. Thus, according to the findings, robot-assisted post-stroke training could be a cost-effective alternative to the conventional rehabilitation service when human manpower is insufficient.

However, almost all positive reports on robot-assisted rehabilitation were obtained through research-oriented clinical trial studies and not in a real clinical service configuration, with the assumption that the positive improvements reported in the trial studies would be naturally carried on into the real services after commercialization. Differences, or even discounts, in the rehabilitation effectiveness during the translation from well-controlled research studies to more flexible services have not yet been intensely evaluated. Actually, the feasibility and effectiveness of rehabilitation robots in the clinical service setting have been questioned when trial-quality management was difficult to achieve in a real long-term service [18–22]. There are several factors that increase the difficulty of head-to-head comparison on the training effectiveness in robot-assisted rehabilitation services with the clinical trials. For instance: (1) In a real service setting, the rehabilitation schedule is relatively flexible with payment from a client. In contrast, trial studies have restricted training schedules (are usually free of charge, or in some cases, participants are even paid for their involvement in the trial); (2) Participant (client) variability is large in the service. In the trials, participant inclusion criteria are usually targeted, and therefore, are difficult to replicate and implement exactly in the service management (particularly in the private sectors) due to the financial sustainability required; (3) In a clinical trial, the participants would usually not be allowed to receive other treatments that might interfere with the prescribed physical program under investigation. However, in a service setting, it is impossible to restrict a client and stop him/her from receiving other physical treatments he/she considers useful. An EMG-driven robotic hand was designed in our previous work, and its rehabilitation effectiveness on the upper limb functions in chronic stroke has been reported by a single group clinical trial [23]. From 2011, the EMG-driven robotic hand service open to local communities has been available in a self-financed university clinic in a private sector. The purpose of this work was to quantitatively evaluate the difference between the rehabilitation effects of an EMG-driven robot hand-assisted upper limb training program conducted as a research trial in a laboratory configuration and as real clinical practice in a private clinic, with minimum disturbance to the routine clinical management and service provided to the clients.

## Methods

### EMG-driven robotic hand

**Fig. 1:**
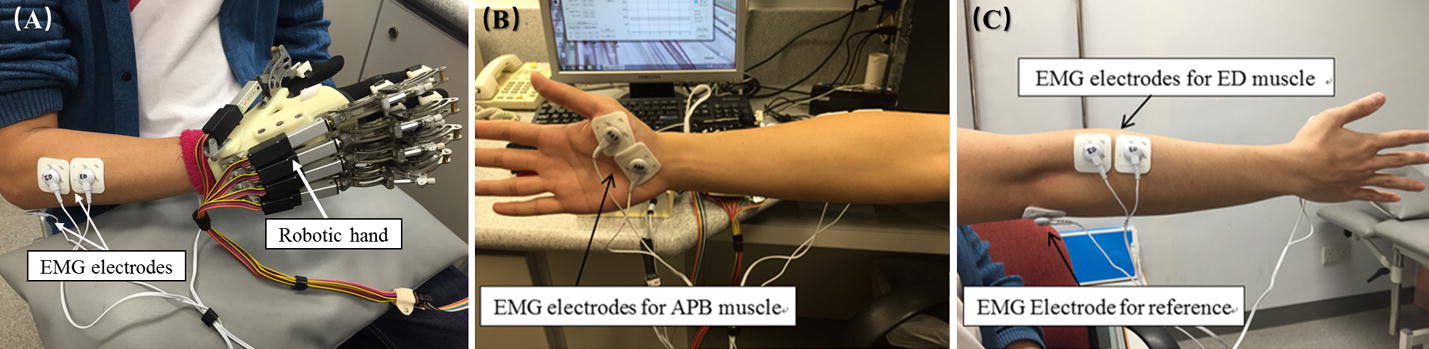
The electromyography (EMG)-driven robotic hand system: **A** The wearable system consisting of a mechanical exoskeleton of the robotic hand and EMG electrodes; **B, C** the illustration of the configuration of the EMG electrodes attached to the extensor digitorum (ED) and abductor pollicis brevis (APB) muscles. The reference electrode was attached on the olecranon

The EMG-driven robotic hand system used in this study is shown in Fig. 1. The system can aid with finger extension and flexion of the paretic limb in stroke patients. The robotic hand consisted of five linear actuators (Firgelli L12, Firgelli Technologies Inc.), and provided individual mechanical assistance to the five fingers [23]. The proximal and distal section of the index, middle, ring and little fingers were rotated around the virtual centers located at the metacarpophalangeal (MCP) and proximal interphalangeal (PIP). The thumb was rotated around the virtual center of its MCP joint. The finger assembly provided two degrees of freedom (DOF) for each finger and offered a range of motion (ROM) of 55° and 65° for the MCP and PIP joints, respectively. The angular rotation speeds of the two joints were set as 22° and 26°/s at the MCP and PIP joints, respectively, during hand open/close.

To facilitate performance of phasic and sequential limb tasks (i.e. hand closing and hand opening), the abductor pollicis brevis (APB) and extensor digitorum (ED) muscles were used as voluntary neuromuscular drives. The APB was selected as the driving muscle in the “hand closing” phase, since the EMG signals from the APB of the paretic limb after stroke are less affected by spasticity and are relatively easier to be controlled than the flexor digitorum (FD) muscle for finger movements in chronic stroke [24]. EMG-triggered control was used in this study. During the training, the threshold level in each motion phase was set at three times the standard deviation (SD) above the EMG baseline in the resting state. In the “hand closing” phase, as soon as the EMG activation level of the APB muscle reached a preset threshold (3 SD above the baseline), the robotic hand would close with a constant speed (22 and 26°/s at the MCP and PIP joints, respectively) and provide mechanical assistance for finger flexion motions. In the “hand opening” phase, once the EMG activation level of the ED muscle reached a preset threshold (3 SD above the baseline), the robotic hand would open with a constant speed (22 and 26°/s at the MCP and PIP joints, respectively). Once the system’s assistance has been initiated, voluntary effort from the patient is not required, and the robot’s assistance will be continuously provided during the entire hand closing and opening phase in the defined ROM.

The EMG signals from the driving muscles captured using EMG electrodes were first amplified 1000 times (preamplifier: INA 333; Texas Instruments Inc., Dallas, TX), sampled at 1000 Hz by using a data acquisition card (DAQ, 6218 NI DAQ card; National Instruments Corp), and filtered by using a band-pass filter in the range 10–500 Hz. After digitization, the EMG signals from the APB and ED muscles were rectified and low-pass filtered (fourth-order, zero-phase forward and reverse Butterworth filter; cut-off frequency, 10 Hz) to obtain an envelope of EMG signals (i.e., the EMG activation level) in the real-time control.

### Clinic versus laboratory

**Fig. 2:**
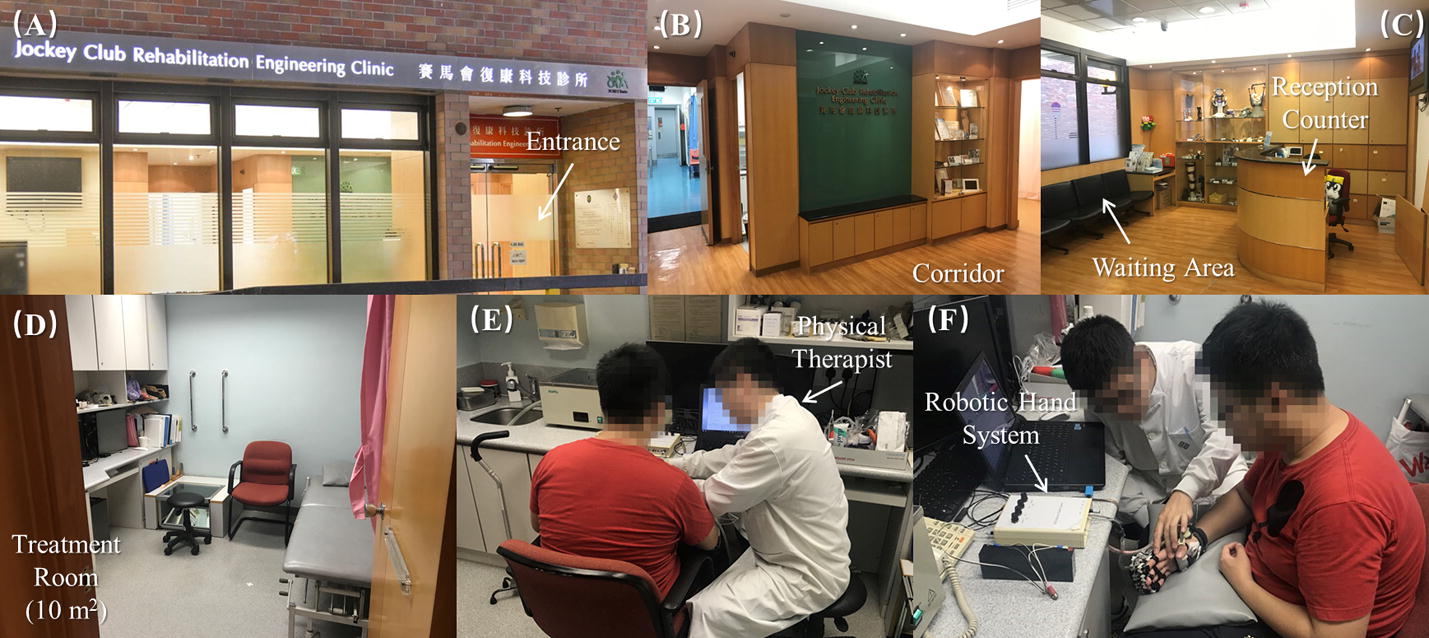
The interior configuration and training setup of the robotic hand training in Jockey Club Rehabilitation Engineering Clinic: **A** Entrance, **B** corridor, **C** waiting area for guests and reception counter, **D** treatment room with estimated area presented by square meter, and **E, F** the training setup of the robotic hand rehabilitation system assisted by a physical therapist

This research concerns a non-randomized, controlled trial comparing two different settings: the clinical service setting under a business environment and the laboratory setting (Table 1). The clinical service was hosted at the University’s Jockey Club Rehabilitation Engineering Clinic (JCREClinic). The JCREClinic provides holistic clinical services, including prostheses, orthoses and robotic rehabilitation training to clients mainly from the local communities. Figure 2 presents the interior configuration of the JCREClinic consisting of a main entrance, reception counter, corridor, waiting area for guests, and treatment rooms. All of the JCREClinic’s consultations and treatments can be arranged by walk-in or scheduled appointments via phone, email or WhatsApp message. The procedure for the robotic hand training service is as follows. First, the client makes an appointment. Then, the client would be invited to a consultation with the physical therapist responsible for the service. In this consultation session, the physical therapist serves the client by reviewing the medical and rehabilitation histories, and then evaluating the motor functions of the upper limb by clinical scores, which would be illustrated in detail later. Following this, the physical therapist helps the client to perform a trial of robotic hand training, including gauging the fit, size, and the possibility of using the target muscles to control the system for the potential treatment. During this stage of the process, the physiotherapist also explains to the client the possible rehabilitation effects, according to the previous results from the trial [23]. Once the client has accepted the robot hand assisted upper limb treatment after the consultation session, a training schedule consisting of 20 sessions (90 min/session) would be arranged by the clinic according to the availabilities of both the physiotherapist and the client with a suggested training frequency of 3–5 sessions/week. In the management of the service, a maximum four sessions/week could be provided to a client. However, the client might re-arrange the schedule later due to other commitments. Each session had a service charge rate of 400 Hong Kong Dollars to be paid after the completion of a session. A client could also quit at any point during the duration of the service without incurring a penalty.

**Table 1:** Clinic versus laboratory

	Clinic	Laboratory
Interior configuration		
Entrance	√	√
Reception counter	√	×
Corridor	√	×
Waiting area	√	×
Treatment room/area	√	√
Appointment		
Walk-in appointment	√	×
Scheduled appointment	√	√
Schedule		
Mutual agreement	√	√
Fixed training intensity	×	√
Accept reschedule	√	×
Contact person		
Reception assistant	√	×
Research staff	×	√
Trainer		
Physical therapist	√	×
Research staff	×	√
Fee	√	×
Withdrawal	√	√

**Fig. 3:**
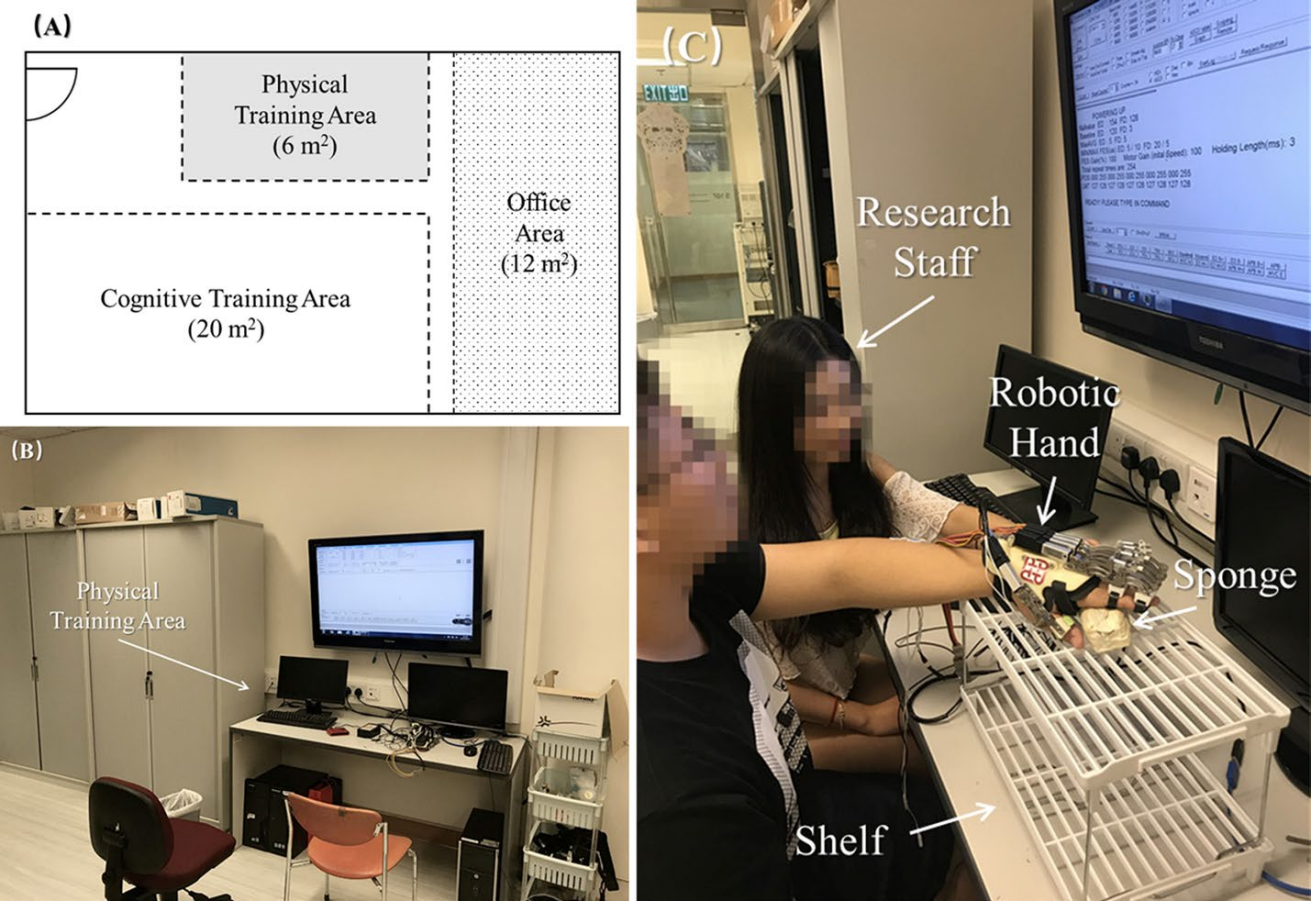
The interior configuration and training setup of the robotic hand training in a neurorehabilitation laboratory: **A** Lab planar graph with estimated area presented by square meter, **B** physical training area, and **C** the training setup of the robotic hand rehabilitation system assisted by a research staff

For the laboratory setting, the EMG-driven robot hand assisted upper limb training was conducted in a neurorehabilitation lab (Fig. 3) in Hong Kong Polytechnic University. The neurorehabilitation lab consists of a physical training area, a cognitive training area, and an office area, and the robotic hand training was performed in the physical training area. The participants who attended the EMG-driven robot hand treatment in the laboratory were not charged for the treatment.

### Participants recruitment

This work was approved by the Human Participants Ethics Sub-Committee of the Hong Kong Polytechnic University. The participants who received their training in the laboratory setting were regarded as the “lab group”, while those in the clinical trial setting were the “clinic group”, and the participants in the two groups were recruited by different approaches. Lab group participants were recruited from the local districts based on the following inclusion criteria [23]: (1) The participants were at least 6 months after the onset of a singular and unilateral brain lesion due to stroke; (2) Both the MCP and PIP joints could be extended to 180° passively; (3) The spasticity during extension at the finger joints and the wrist joint was lower than or equal to 3 as measured by the Modified Ashworth Scale (MAS) [25]; (4) There should be detectable voluntary EMG signals (i.e. the signal amplitude should exceed 3 SD above the mean of the baseline) from the target muscles in the paretic side of the participants; (5) The participants also had sufficient cognition to follow the experimental instructions as assessed by the Mini-Mental State Examination (MMSE > 21). In addition to these factors, lab group participants were also told that they could not attend other upper limb physical treatments during the robotic hand training, otherwise they would be dropped from the study. Before initiating treatment, all recruited participants had to submit their written consent.

**Fig. 4:**
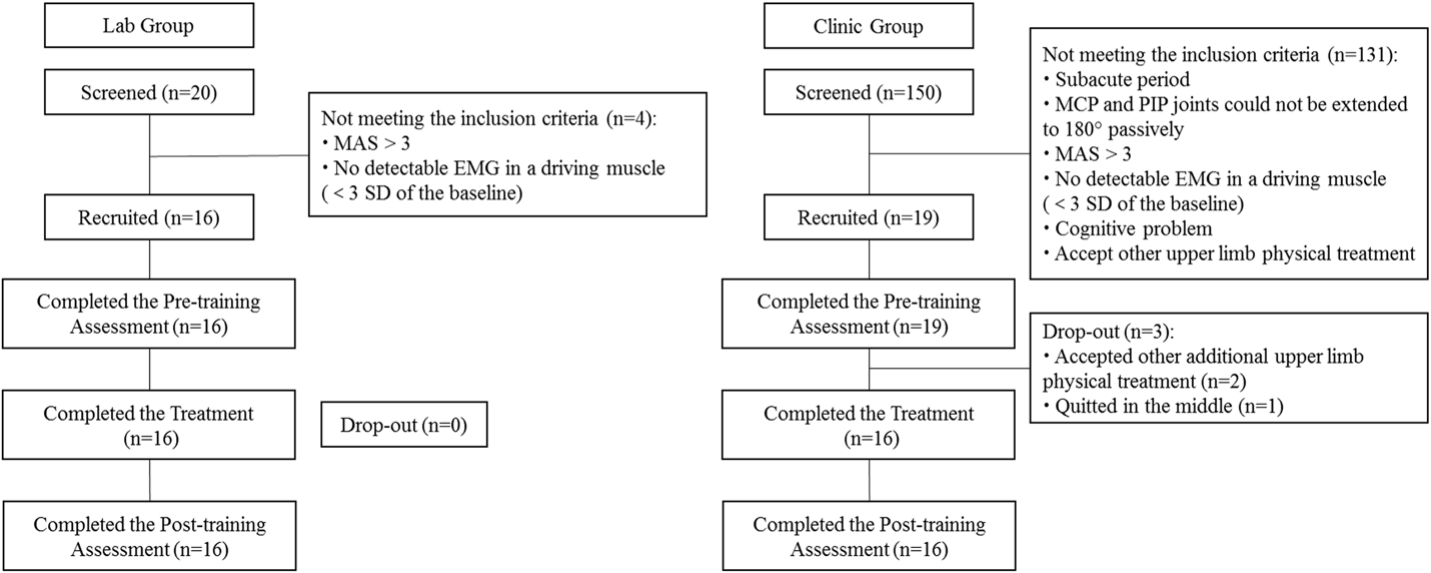
The consolidated standards of reporting trials flowchart of the experimental design

Participants in the clinic group were recruited from a pool of clients scheduled for robotic hand rehabilitation in the JCREClinic. Clients were screened in the JCREClinic and potential participants were those who possessed the upper limb motor deficits that satisfied the same inclusion criteria as for the lab group. Then, the clients who showed the interest in participation and agreed not to receive other upper limb treatment during the training period were recruited in the study. Figure 4 illustrates the Consolidated Standards of Reporting Trials flowchart of the training program.

### Training protocols

In this study, all participants attended the 20-session robotic hand assisted upper limb training. In each session, a participant was instructed to conduct the repetitive upper limb motions including hand grasp and release motions, and lateral task training and vertical task training. In the lateral task, each participant was instructed to grasp a sponge (thickness 5 cm, weight 30 g) that was placed on one side of a table near the paretic side of the participant, transport the sponge 50 cm horizontally, release it, grasp it again, and return it to the starting point. In the vertical task, each participant was instructed to grasp the sponge on the midline of the lower layer of a shelf, lift it through a vertical distance of 17 cm, place it on the midline of the upper layer of the shelf, grasp it again, and place it back on the starting point. The procedures have been fully detailed in our previous study [23].

The key training program differences between the clinic and the laboratory groups were the training pace and frequency per week, and the interaction between the treatment operator and the participant. In the clinic group, the recruited participants (clients) received the robotic hand training in a treatment room of the JCREClinic by a physiotherapist in a one-to-one manner, with a duration of 90 min for each session. The training frequency for those in the clinic group was negotiable with a maximum of four sessions/week. However, the final averaged training frequency in the clinic group was 2.25 sessions/week, with a range of 1–3 sessions/week, due to the re-arrangements raised in the middle in this study. In each session, a participant in the clinic group had a relatively flexible training pace, i.e., the participant could stop the practice for a rest of 5 min whenever needed to avoid significant fatigue. During the rest, verbal communication between physiotherapist and the participant, and encouragement from the therapist were delivered. In a 90-min training session, it was observed that the participants in the clinic group could gradually increase the accumulated practicing time from less than 45 min to more than 60 min (on average) throughout the treatment process.

By comparison, each participant in the lab group was invited to attend the robotic hand training in the laboratory by a research assistant in the project with a training frequency of four sessions/week, over a 5-week (consecutive) period. One 10-min break was given for every 20 min of training to reduce the muscle fatigue. The accumulated practicing time in a session was 60 min, as in our previous trial [23].

### Outcome evaluations

The motor functional improvements in the upper limb of the participants were reviewed using a series of clinical scores by an assessor who was blinded to the protocol of the study. In this study, the applied assessments included the Fugl-Meyer Assessment [26] [FMA with the full score of 66 for the upper limb assessment was further divided into shoulder/elbow (42/66) and wrist/hand (24/66)], MAS [25] on the flexors related to the fingers, wrist and elbow, Action Research Arm Test (ARAT) [27], and Functional Independence Measure (FIM) [28]. The FMA evaluates the motor function impairment in voluntary limb movements. The MAS measures the resistance during passive muscle stretching and indicates the muscular spasticity, mainly in the flexors. The ARAT assesses the upper limb voluntary functions with a focus on the finger activities. The FIM was used to rate the basic quality of daily living activities (ADLs) for patients with stroke.

### Statistics

The varying demographic characteristics of the participants between the two groups were assessed by the independent *t* test or the Fisher exact test. The baselines of the clinical scores for the two groups were compared by independent t-test with an insignificant statistical difference (P > 0.05) on the primary clinical assessments (i.e., pre-assessments on FMA). One-way analysis of covariance (ANCOVA) was then used to evaluate the group differences of the post-training clinical assessments by taking the pre-assessment as a covariate. Following this, paired t-test was conducted to investigate the intragroup difference of the two groups at different time points both before and after the training. Furthermore, the changes of each clinical assessment after the treatments were also compared between the groups by independent t-test. The levels of statistical significance were indicated at 0.05, 0.01, 0.001 in this study.

## Results

For the lab group, 20 stroke patients were screened, and from these, 16 participants were recruited. For the clinic group, 150 stroke clients were screened in the JCREClinic; 19 participants fulfilled the inclusion criteria and were therefore, recruited into the clinic group. In total, three participants dropped out in the clinic group: two, because they attended other additional upper limb physical treatment during the training, and one, who decided to quit in the middle of the training. Therefore, a total of 32 participates completed the EMG-driven robotic hand assisted upper limb training, either in a clinical trial study (n = 16) or in a clinical service (n = 16). The recruited participants’ demographic data are presented in Table 2. No statistical differences were observed between the groups in terms of age, gender, side of stroke, type of stroke, employment, and onset time. Twenty-eight participants quit their employment, and four participants in the clinic group were still working during the period of the study. Higher training sessions per week (P < 0.001, independent t-test, Table 2) can be observed in the lab group compared with the clinic group.

**Table 2:** Demographic characteristics of the participants

Characteristics	Clinic group (n = 16)	Lab group (n = 16)	P value
Age in years (mean ± SD)^a^	53.50 ± 13.08	53.06 ± 10.27	0.917
Gender (male/female)^b^	8/8	12/4	0.273
Stroke side (right/left)^b^	9/7	10/6	1.000
Type of stroke (ischemic/hemorrhagic)^b^	10/6	10/6	1.000
Employment (working/not working)^b^	4/12	0/16	0.101
Times since stroke in years (mean ± SD)^a^	3.16 ± 1.85	5.53 ± 4.30	0.052
Training sessions per week (mean ± SD)^a^	2.25 ± 0.58	4.00 ± 0.00	0.000***

Difference with statistical significance is marked with ‘*’ (P < 0.05, independent t-test). Significant levels are indicated as, 1 asterisk for < 0.05, 2 asterisks for ≤ 0.01, and 3 asterisks for ≤ 0.001

^a^Test for independent samples

^b^Fisher’s exact test

Table 3 details the comparisons between the two groups on the clinical scores before the training. Significant inter-group differences on pre-clinical assessment were found in the MAS elbow (P = 0.044, EF = 0.75, independent t-test, Table 3) and ARAT (P = 0.041, EF = 0.76, independent t-test, Table 3). There was no significant difference on the pre-clinical assessment between the two groups in the MAS finger, MAS wrist, FMA, and FIM (P > 0.05, independent t-test, Table 3).

**Table 3:** The pre-clinical assessments of each group

Clinical score	Clinic Group	Lab group	P values (Cohen’s d)
FMA_full score_	13.75 ± 11.44	17.50 ± 15.26	0.438 (0.28)
FMA_shoulder/elbow_	10.31 ± 8.14	12.44 ± 10.48	0.527 (0.23)
FMA_wrist/hand_	3.44 ± 4.18	5.06 ± 5.50	0.354 (0.33)
ARAT	3.81 ± 8.30	11.69 ± 12.18	0.041 (0.76)*
FIM	56.63 ± 9.25	58.50 ± 14.09	0.660 (0.16)
MAS_finger_	1.70 ± 0.76	1.34 ± 1.08	0.279 (0.39)
MAS_wrist_	1.65 ± 0.95	1.10 ± 0.66	0.066 (0.67)
MAS_elbow_	1.91 ± 0.74	1.21 ± 1.10	0.044 (0.75)*

The mean and standard deviations (SD) for each measurement of the pre-clinical assessments, and the probabilities with the estimated effect sizes of the statistical analyses. Intergroup differences with statistical significance are marked with ‘*’ (P < 0.05, independent t-tests)

*FMA* Fugl-Meyer Assessment, *ARAT* Action Research Arm Test, *FIM* functional independence measure, *MAS* Modified Ashworth Scale

**Fig. 5:**
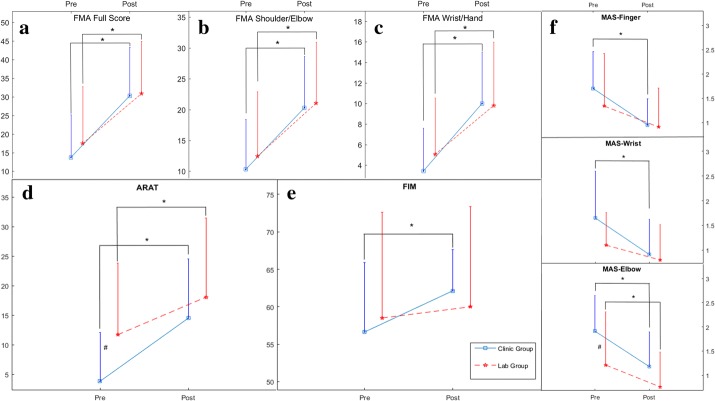
The clinical scores (evaluated before the first and after the 20th training session) of the participants in both clinic group and lab group: **a** Fugl-Meyer Assessment (FMA) full scores, **b** FMA shoulder/elbow scores, **c** FMA wrist/hand scores, **d** Action Research Arm Test (ARAT) scores, **e** Functional Independence Measure (FIM), and **f** Modified Ashworth Scale (MAS) scores at the fingers, the wrist and the elbow, presented as mean value with 2-time SE (error bar) in each evaluation session. The solid lines are for the clinic group, and the dashed lines are for the lab group. The significant intragroup difference is indicated by “*” (p < 0.05, paired t-test), and “#” is used to indicate the significant intergroup difference (p < 0.05, independent t-test)

Figure 5 compares the two groups’ clinical scores of the FMA, ARAT, FIM, and MAS evaluated before the first training session (pre-training assessment) and after the last training session (post-training assessment). The values of each clinical assessment of the two groups have been summarized in Table 4. In the clinic group, significant increases were observed in the clinical scores of the FMA full score (P < 0.001, EF = − 1.45, paired t-test, Table 4), FMA shoulder/elbow (P < 0.001, EF = − 1.35, paired t-test, Table 4), FMA wrist/hand (P < 0.001, EF = − 1.36, paired t-test, Table 4), ARAT (P < 0.001, EF = − 1.22, paired t-test, Table 4), and FIM (P = 0.004, EF = − 0.86, paired t-test, Table 4); while significant decreases were obtained in the MAS finger (P < 0.001, EF = 1.12, paired t-test, Table 4), MAS wrist (P = 0.001, EF = 0.97, paired t-test, Table 4) and MAS elbow (P = 0.001, EF = 1.08, paired t-test, Table 4). For the lab group, significant increases were observed in the scores of the FMA full score (P < 0.001, EF = − 1.46, paired t-test, Table 4), FMA shoulder/elbow (P < 0.001, EF = − 1.18, paired t-test, Table 4), FMA wrist/hand (P < 0.001, EF = − 1.35, paired t-test, Table 4) and ARAT (P < 0.001, EF = − 1.44, paired t-test, Table 4). Significant decreases were only obtained in the MAS elbow (P = 0.013, EF = 0.71, paired t-test, Table 4). However, no significant group differences were observed on scores in the post-assessments (P > 0.05, one-way ANCOVA, Table 4). As the interaction between the pre-clinical scores and group factor of the FIM score was significant (P < 0.05), one-way ANCOVA could not be used to evaluate the post-clinical scores of FIM between the two groups. Therefore, the variations of the clinical scores were further used to evaluate the intergroup comparison particularly on the FIM scores.

**Table 4:** The clinical assessments of each group

Assessment	Group	Mean value (95% confidence interval)		Paired t test	1-way ANCOVA
		PRE	POST	P (Cohen’s d)	P (Partial η2)
FMA_full score_	Clinic	13.75 (8.09–19.41)	30.31 (23.88–36.75)	0.000 ( − 1.45)***	0.550 (0.012)
	Lab	17.50 (9.95–25.05)	30.88 (23.92–37.83)	0.000 ( − 1.46)***	
FMA_shoulder/elbow_	Clinic	10.31 (6.28–14.34)	20.31 (16.18–24.44)	0.000 ( − 1.35)***	0.782 (0.003)
	Lab	12.44 (7.25–17.63)	21.06 (16.16–25.97)	0.000 ( − 1.18)***	
FMA_wrist/hand_	Clinic	3.44 (1.37–5.51)	10.00 (7.52–12.48)	0.000 ( − 1.36)***	0.333 (0.032)
	Lab	5.06 (2.34–7.78)	9.81 (6.75–12.87)	0.000 ( − 1.35)***	
ARAT	Clinic	3.81 (− 0.30 to 7.92)	14.50 (9.56–19.44)	0.000 ( − 1.22)***	0.175 (0.063)
	Lab	11.69 (5.66–17.72)	18.06 (11.43–24.69)	0.000 ( − 1.44)***	
FIM	Clinic	56.63 (52.05–61.20)	62.13 (59.41–64.84)	0.004 ( − 0.86)**	Nil
	Lab	58.50 (51.53–65.47)	60.00 (53.39–66.61)	0.161 ( − 0.37)	
MAS_Finger_	Clinic	1.70 (1.32–2.08)	0.95 (0.68–1.22)	0.000 (1.12)***	0.622 (0.009)
	Lab	1.34 (0.81–1.87)	0.91 (0.52–1.31)	0.085 (0.46)	
MAS_Wrist_	Clinic	1.65 (1.18–2.12)	0.91 (0.56–1.26)	0.001 (0.97)***	0.443 (0.020)
	Lab	1.10 (0.77–1.43)	0.80 (0.45–1.15)	0.075 (0.48)	
MAS_Elbow_	Clinic	1.91 (1.54–2.28)	1.18 (0.82–1.53)	0.001 (1.08)***	0.892 (0.001)
	Lab	1.21 (0.77–1.66)	0.76 (0.40–1.12)	0.013 (0.71)*	

The mean and 95% confidence intervals for each measurement of the clinical assessments, and the probabilities with the estimated effect sizes of the statistical analyses. Intragroup differences with statistical significance are marked with ‘*’ (“*” for paired t-tests). Significant levels are indicated as, 1 asterisk for < 0.05, 2 asterisks for ≤ 0.01, and 3 asterisks for ≤ 0.001

**Fig. 6:**
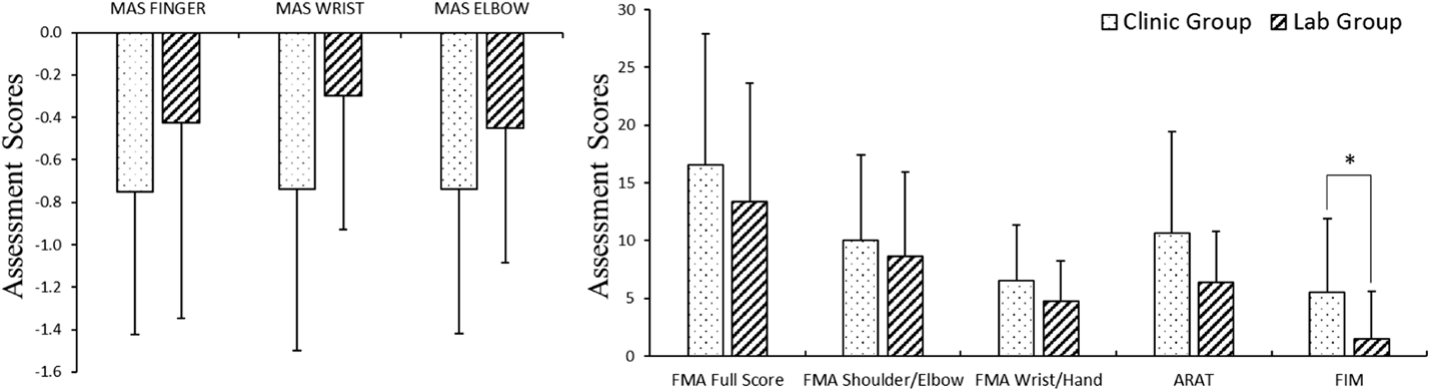
The changes of each clinical assessment after the treatments in both clinic and lab groups: Fugl-Meyer Assessment (FMA) full scores, FMA shoulder/elbow, FMA wrist/hand, Action Research Arm Test (ARAT), Functional Independence Measure (FIM) and Modified Ashworth Scale (MAS) scores at the fingers, the wrist and the elbow, presented as mean value with 2-time SE (error bar) in each evaluation session. The significant difference is indicated by “*” (P < 0.05, independent t-test)

Figure 6 lists the changes of each clinical assessment for the two groups following their respective treatments. The detailed values and the statistical results of the comparison have been summarized in Table 5. Compared with the lab group, significant higher variations in the clinic group were observed on the FIM scores (P = 0.043, EF = 0.75, independent t-test, Table 5). No significant variations of the clinical scores between the two groups were achieved in the MAS, FMA and ARAT (P > 0.05, independent t-test, Table 5).

**Table 5:** The changes of each clinical assessment of each group

Clinical score	Clinic group	Lab group	P value (Cohen’s d)
FMA_full score_	16.56 ± 11.38	13.38 ± 10.29	0.390 (0.29)
FMA_shoulder/elbow_	10.00 ± 7.39	8.63 ± 7.29	0.600 (0.19)
FMA_wrist/hand_	6.56 ± 4.83	4.75 ± 3.51	0.234 (0.43)
ARAT	10.69 ± 8.73	6.38 ± 4.44	0.088 (0.62)
FIM	5.50 ± 6.41	1.50 ± 4.07	0.043 (0.75)*
MAS_finger_	− 0.75 ± 0.67	− 0.43 ± 0.92	0.263 (0.40)
MAS_wrist_	− 0.74 ± 0.76	− 0.30 ± 0.63	0.226 (0.63)
MAS_elbow_	− 0.74 ± 0.68	− 0.45 ± 0.63	0.086 (0.44)

The mean and standard deviations (SD) for the changes of each clinical assessment, and the probabilities with the estimated effect sizes of the statistical analyses. Intergroup difference with statistical significance is marked with ‘*’ (“*” for independent t-test)

## Discussion

After 20 sessions of upper limb training assisted by EMG-driven robotic hand, improved clinical scores denoting enhanced motor function were observed in all participants, and were recorded in the shoulder, elbow and finger after the treatments.

The significant increase in the FMA shoulder/elbow score after the training in both groups indicated that robotic hand training could improve the motor control at the joints of the shoulder and elbow with equivalent training effectiveness. Although no specific robotic system was applied to the joints of the elbow and shoulder in this study, the increase in the FMA shoulder/elbow scores after robotic hand training can still be observed. Possible reasons for this are as follow: (1) The involvement of other joints in the training tasks might be beneficial to the whole upper limb [29]; in this study, the participants’ shoulder-related and elbow-related muscles were practiced during the training process via the lateral task training and the vertical task training; and (2) the adjacent proximal joint would be simultaneously improved when the muscle around the joint was trained, as indicated by our previous studies [30, 31]. Hence, the wrist training might lead to elbow improvement [31], and the elbow training might lead to improved shoulder function [30]. As proximal to distal gradient of motor deficit being absent [32], this result suggests that task-oriented whole upper limb training is a more beneficial method than joint-per-joint rehabilitation, which is consistent with research by Susanto et al. [29] and Oujamaa et al. [33]. Both groups’ significant increase in the FMA hand/wrist scores also demonstrated that this study’s EMG-driven robotic hand could assist stroke patients with improving motor function in their wrists and hands, with a comparable achievement between the clinic group and the lab group. The ARAT score is mainly to evaluate the finger movements as well as grasping, gripping, and pinching movements. The significant increased score in the ARAT scores in both group after the training suggested the improved finger coordination for fine precision grasping and joint stability of the fingers, which was consistent with the increased FMA wrist/hand score.

When comparing the functional improvements between the two groups, the effectiveness of the robotic hand applied in the private clinic is statistically equivalent to that found in the research laboratory, where the clinic service group improved more with the lower training frequency compared with the lab group. Furthermore, the improvement on the ADLs indicated by the FIM scores in the clinic group was significantly better than the improvement on the ADLs achieved in the lab group. The FIM score is mainly used to rate the basic quality of daily living activities for patients with stroke. The significantly increased FIM scores in the clinic group reveal that the EMG-driven robotic hand was effective in improving the independence of ADLs for chronic stroke patients in the clinic group. However, there was no significant improvement in the FIM scores obtained in the lab group after the robotic hand training. In addition, the notable decrease in the MAS score at elbow, wrist and fingers for participants in the clinic group indicated that robotic hand training could improve the muscle coordination and joint stability of the proximal and distal joints not only during hand grasp and release motions, but also during arm reaching motions. For participants in the lab group, however, the significant decrease in the MAS scores was only observed on the elbow joint, and no noteworthy decrease in the MAS scores on the fingers and wrist were recorded for the lab group following the robotic hand training.

Thus, the question is why the clinic group achieved better ADLs and released muscle tone on hand despite the lower training frequency per week. One possible explanation for this is that the participants in the clinic group performed daily practice by themselves besides the treatment in the clinic. In the clinical service, the physical therapist always suggested and encouraged stroke patients to practice the hand grasp and release motion and arm reaching motions every day with the purpose to generalize the learnt motor skill in the daily functions. Those stroke patients actively followed the professional suggestions and performed daily living activities, such as self-feeding, dressing and bathing using their affected limb. In contrast, however, the research staff did not expressly suggest that the stroke patients in the laboratory practice by themselves in daily activities. In this study, the significant decrease in the MAS wrist in the clinic group after the robotic hand training suggested the release of spasticity in the wrist joint for these participants; no similar results were observed in the lab group. However, the joints of wrist were fixed on the palm-wrist module (shown in Fig. 1), and no specific tasks were assigned to the wrist joints in this study; therefore, the decreased spasticity in the wrist joint noted for participants in the clinic group may not be directly attributed to the robotic hand training, but instead may be due to the stroke patients’ own self-practice. In addition, compared with the lab group, the significantly improved FIM scores in the clinic group may also be a result of the participants’ additional self-practice with daily living activities.

Another unique aspect of the clinical service is the flexible training pace (also called voluntary exercise). During the robotic training, the participant could voluntarily control the training pace by deciding to rest whenever they needed, or by continuing to perform the robotic training without a rest. The average accumulated practicing time per session ranged from less than 45 min to more than 60 min throughout the whole training program. For example, in the first few training sessions, participants in the clinic group usually frequently asked for a rest after every 5 min of training. When the participants were familiar with the training program, they could gradually increase their practicing time to around 60 min per session. For those participants who could perform well with the robotic hand training, their total practicing time per session might even exceed 60 min, and they also claimed that they could practice more if there was no time limited. However, in the lab group, the participants took a 10-min break for every 20 min of training, with a 60-min accumulated practice time per session. Although little research has been conducted on the effect of voluntary exercise in stroke rehabilitation for human beings, some studies based on the post-stroke mice model have demonstrated that better results are achieved through voluntary exercise than through forced exercise. For instance, Ke et al. [34] used three approaches involving the voluntary exercise of wheel running (V-Ex), forced exercise of treadmill running (F-Ex), and involuntary exercise of FES (I-Ex) to train post-stroke rats. The V-Ex rats were housed individually in a cage with a running wheel assembled and left to freely run of their own accord, similar to the flexible training in our clinic group. However, F-Ex rats were forced to run on the motor-driven treadmill for a total of 30 min every day, which was like the fixed training in our lab group. The results revealed that the voluntary exercise was the most effective training style in facilitating motor recovery, while the forced exercise group achieved the least motor recovery, consistent with the findings of Lin et al. [35]. Thus, this may be the reason for the larger improvements achieved by the clinic group in this study, and is therefore, one potential avenue for further study on post-stroke rehabilitation for human beings.

Motivation has been regarded as the key to stroke rehabilitation and plays an important role in determining recovery outcomes [36]. It is widely believed that patients with high motivation can achieve better outcome compared with those patients with less enthusiastic for treatment [37, 38]. In this study, we observed that the stroke patients in the clinical service had higher motivation than those who were in the laboratory. It has been found that motivation is a multi-determined phenomenon involving various aspects, such as patient characteristics (including personality traits, anxiety, age, socio-economic status), social factors (including the qualities of the practitioner and patient-practitioner interaction) and rehabilitation environment [39, 40]. Thus, the higher socio-economic status in the clinic group compared with that of the lab group may have influenced the patients’ motivations. For example, there were four stroke patients in the clinic group who were still employed and therefore, were strongly motivated to regain functional recovery; however, in the lab group, the participants had all quit their jobs, might not have confidence in their own abilities to perform everyday activities, and had low motivation to achieve functional recovery. The qualities of the practitioner and patient-practitioner interaction are also linked with the formation of patient motivation [41]. A practitioner exhibiting strong confidence in the treatment with good communication skills can improve patients’ positive motivation, while a neutral or uncertain attitude may have little or even a negative impact on the patients [42, 43]. Therefore, a professional physical therapist who wears a doctor’s overall and can provide professional rehabilitation guidelines in a clinic setting may subconsciously enhance the positive motivation of the patients in the clinic group. Furthermore, the treatments delivered in different rehabilitation environments may affect a patient’s belief or faith in the treatment they receive. According to a qualitative analysis of stroke professionals’ attitudes [40], it was pointed out that a stimulating rehabilitation environment with a well-maintained treatment room is a positive determinant of motivation. Hence, positive patients’ motivation requires an encouraging environment and good interaction between the therapists and the patients. Some other studies also suggested that training devices which can provide rewarding schemes in a gaming environment had the ability to enhance the motivation of patients [44, 45]. However, how to optimally apply motivational therapy into the rehabilitation to achieve better training outcomes is still unclear and need to be further investigated [46].

It was noticed that the ARAT and MAS Elbow scores for the clinic group were significantly lower than the lab group. However, there was no significant difference between the groups in the pre-assessment for the other clinical scores. It might imply that the upper limb motor function of the clinic group could be lower than the lab group during the admission. In the post-assessment, the rehabilitation outcomes of the clinic group were comparable or even better (e.g., FIM) than the lab group. It might imply that the robotic hand training was more effective for those severely injured patients. The main limitation of this study was the small sample size. Large scale clinical trial with stratified randomization in multi-centers will be conducted in our future work to further validate the rehabilitation effectiveness of the device assisted post-stroke rehabilitation.

## Conclusion

The improvements of the EMG-driven robotic hand training obtained in a clinical service were similar to the effectiveness of the same robotic training carried out in a research environment. A higher independence in the daily living activities and more effective release in muscle tones was achieved in the clinic group than the lab group. The potential better outcomes in the clinic group may due to the flexible training, self-exercise, and higher motivation. This study provides additional support for the role of robotics training in the clinic service for post-stroke patients after translation from the research laboratory. It has further demonstrated the feasibility and efficacy of robotic hand-assisted upper limb therapy with a flexible service in improving the distal function, which further translates to improvements in the elbow and shoulder.
